# Hypermobility, immune dysfunction and dysautonomia cluster in ADHD

**DOI:** 10.1192/j.eurpsy.2025.189

**Published:** 2025-08-26

**Authors:** J. Kustow

**Affiliations:** Director of Education, UK Adult ADHD Network, London, United Kingdom

## Abstract

**Abstract:**

Emerging research reveals a striking overlap between ADHD, hypermobility syndromes, immune dysfunction, and autonomic dysregulation. Studies suggest that approximately half of individuals with ADHD are hypermobile, while ADHD is significantly over-represented in those with hypermobility syndromes, such as Hypermobile Ehlers-Danlos Syndrome (hEDS) and Hypermobility Spectrum Disorder (HSD). Unlike benign joint hypermobility, these syndromes involve multisystem pathology, often accompanied by dysautonomia (e.g., postural orthostatic tachycardia syndrome, POTS), mast cell activation disease (MCAD), and autoimmune conditions.

This **‘**somatic super-syndrome’ encompasses many of ADHD’s under-recognised somatic comorbidities, including hypermobility, allergy and autoimmunity, POTS, fatigue and pain syndromes (Chronic Fatigue Syndrome and Fibromyalgia Syndrome), and sensory processing issues, amongst others. A small but growing body of evidence suggests that mast cells - **‘**first responder’ immune cells involved in allergic and inflammatory responses - play a critical role in neurocognitive function and that their excessive activation has been associated with a range of neurological and psychiatric/neurodevelopmental conditions. Could a similar issue with aberrant mast cell activation be contributing to the pathophysiology of ADHD.

Dr. James Kustow, consultant psychiatrist and adult ADHD specialist, will present the latest insights into this complex interplay between connective tissue dysfunction, chronic low-level inflammation, and autonomic dysregulation. He will explore how these factors may, in some, drive an ADHD-like syndrome and discuss emerging research on neuroimmune mechanisms linking ADHD with inflammatory disorders such as infections, asthma, rhinitis, and food allergies.

By illuminating these connections and reviewing the available evidence, this presentation will encourage a broader, integrative approach to understanding and managing ADHD. It will also consider how future research might inform novel therapeutic strategies targeting immune dysregulation and autonomic dysfunction in ADHD.

**Disclosure of Interest:**

J. Kustow Consultant of: I have done some consultancy work for a couple of pharmaceutical companies but not for over 3 years, Speakers bureau of: I have previously spoken at and chaired events organised by pharmaceutical companies (but I dont speak on the subject of ADHD medication).

